# Resistance to commonly used insecticides and phosphine fumigant in red palm weevil, *Rhynchophorus ferrugineus* (Olivier) in Pakistan

**DOI:** 10.1371/journal.pone.0192628

**Published:** 2018-07-19

**Authors:** Waqas Wakil, Muhammad Yasin, Mirza Abdul Qayyum, Muhammad Usman Ghazanfar, Abdullah M. Al-Sadi, Geoffrey O. Bedford, Yong Jung Kwon

**Affiliations:** 1 Department of Entomology, University of Agriculture, Faisalabad, Pakistan; 2 College of Agriculture, Bahauddin Zakariya University Bahadur Sub-Campus, Layyah, Pakistan; 3 Department of Entomology, Muhammad Nawaz Shareef University of Agriculture, Multan, Pakistan; 4 Department of Plant Pathology, College of Agriculture, University of Sargodha, Pakistan; 5 Department of Crop Sciences, College of Agricultural and Marine Sciences, Sultan Qaboos University, AlKhoud, Oman; 6 Department of Biological Sciences, Macquarie University, NSW, Australia; 7 College of Agriculture and Life Sciences, Kyungpook National University, Daegu, South Korea; Northwest A&F University, CHINA

## Abstract

The red palm weevil *Rhynchophorus ferrugineus* (Olivier) is an important pest of date palms in many regions of the world. This paper reports the first survey of insecticide resistance in field populations of *R*. *ferrugineus* in Pakistan which were collected from seven date palm growing areas across Punjab and Khyber Pakhtunkhwa (KPK) provinces, Pakistan. The resistance was assessed by the diet incorporation method against the formulated commonly used chemical insecticides profenophos, imidacloprid, chlorpyrifos, cypermethrin, deltamethrin, spinosad, lambda-cyhalothrin and a fumigant phosphine. Elevated levels of resistance were recorded for cypermethrin, deltamethrin and phosphine after a long history of insecticide use in Pakistan. Resistance Ratios (RRs) were 63- to 79-fold for phosphine_,_ 16- to 74-fold for cypermethrin, 13- to 58-fold for deltamethrin, 2.6- to 44-fold for profenophos, 3- to 24-fold for chlorpyrifos, 2- to 12-fold for lambda-cyhalothrin and 1- to 10-fold for spinosad compared to a susceptible control line. Resistant *R*. *ferrugineus* populations were mainly found in southern Punjab and to some extent in KPK. The populations from Bahawalpur, Vehari, Layyah and Dera Ghazi Khan were most resistant to chemical insecticides, while all populations exhibited high levels of resistance to phosphine. Of the eight agents tested, lower LC_50_ and LC_90_ values were recorded for spinosad and lambda-cyhalothrin. These results suggest that spinosad and lambda-cyhalothrin exhibit unique modes of action and given their better environmental profile, these two insecticides could be used in insecticide rotation or assist in phasing out the use of older insecticides. A changed pattern of both insecticides can be used sensibly be recommended without evidence of dose rates and frequencies used.

## Introduction

Red Palm Weevil *Rhynchophorus ferrugineus* (Olivier) (Coleoptera: Curculionidae) is one of the most damaging insect pests of almost all kind of palms worldwide [1, 2, 3]. The larval stages damage host plants, usually remain in the lower 1 m of the tree trunk and can complete several generations within the same host [4, 5, 6]. So far it is established in 50% of date growing and 15% of coconut producing countries [1]. Early infestation cannot be detected and goes unnoticed. By the time the farmer recognizes the problem, the growing point (or cabbage) of the palm could have already been damaged with significant numbers of larvae developing inside the trunk generally resulting in the death of the palm [1, 7, 8,].

Synthetic insecticides have been used as the principal control tactic of this pest. Insecticidal treatments with fumigants, soil drench, frond axil filling, trunk injections, wound dressing and crown drenching being the main strategies for *R*. *ferrugineus* control [9]. The efficacy of different chemical insecticides using various application methods singly and in mixtures has been studied [10, 11, 12, 13, 14, 15, 16, 17, 18].

In Pakistan, application of insecticides and fumigants on date palms against *R*. *ferrugineus* has a long history, but published records are lacking. Ten different insecticides were evaluated against *R*. *ferrugineus* during field trials and data showed fipronil, spirotetramat, chlorpyrifos and methidathion the most effective [19]. In Pakistan the effectiveness of commercially used insecticides has been questioned by the date palm growers. Reduced effectiveness of these insecticides may be due to the development of resistance, although, the mechanism of resistance against commonly used insecticides is poorly understood. A limited exploration of resistance development in *R*. *ferrugineus* is the focus of the current investigation. We selected seven commonly used insecticides and phosphine (aluminium phosphide), and assessed their effectiveness against geographically distinct populations of *R*. *ferrugineus* collected from seven different areas of Punjab and Khyber Pakhtunkhwa (KPK), Pakistan.

## Materials and methods

### *Rhynchophorus ferrugineus* collection and rearing

**Fig 1:**
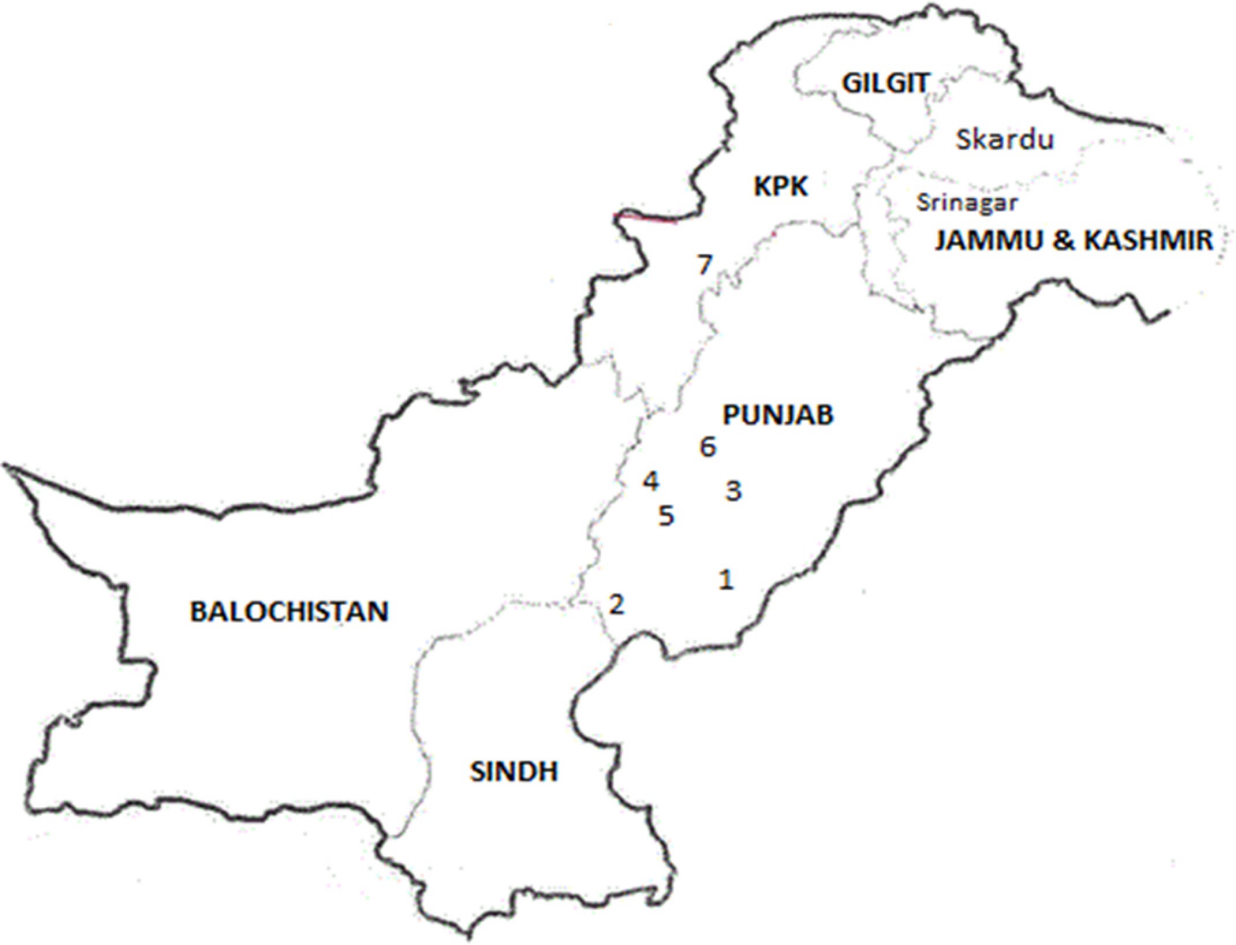
Location of seven sampling sites for *R*. *ferrugineus* field populations in Punjab and KPK, Pakistan 1: Bahawalpur; 2: Rahim Yar Khan; 3: Vehari; 4: Dera Ghazi Khan; 5: Muzaffargarh; 6: Layyah; 7: Dera Ismail Khan.

*Rhynchophorus ferrugineus* at different developmental stages were collected from fallen and infested date palm trees from various areas of Punjab and KPK during 2014–2015 (Fig 1). The areas were selected on the basis of long history of date palm cultivation and long term use of insecticides and phosphine. The application of insecticides here was regular and frequent but the palms had not been sprayed before insect collection. Adults, larvae and pupae were collected into separate plastic jars and brought back to the laboratory. In the laboratory larvae were provided with sugarcane (*Saccharum officinarum* L.; Poales: Poaceae) stems for food and as pupation site, while adults were offered shredded sugarcane pieces for both food and as an oviposition substrate in plastic boxes. Pupae were kept in separate boxes for adult emergence in incubators (Sanyo Corporation, Japan) at 27±2°C, 60±5 RH and photoperiod of 12:12 (D:L) hours. On emergence adults were transferred to jars for feeding and mating. The colonies were maintained in plastic boxes (15×30×30 cm) having a lid whose center (8-cm) diameter covered with mesh wire gauze (60 mesh) for aeration. Rearing was carried out at the Microbial Control Laboratory, Department of Entomology, University of Agriculture, Faisalabad, Pakistan. The adult food was changed after every three days and the removed sugarcane pieces were kept in separate jars for eggs to hatch. After egg hatch neonate larvae were transferred to sugarcane pieces for feeding until freshly molted 4^th^ instar larvae were recovered. The laboratory strain which was used as the reference strain had been reared in the laboratory for over 25 generations since 2009 without exposure to insecticides and will be maintained for future research. Preliminary laboratory bioassays it showed highly susceptible to tested insecticides, being as susceptible as those used earlier [20].

### Test chemicals

For bioassays, commercial formulations of Curacron^®^ (profenophos, 500 g/litre, 500 EC; Syngenta Pakistan Ltd., Karachi, Pakistan); Confidor^®^ (imidacloprid, 700g/kg, 70 WG; Bayer Crop Sciences, Pakistan Pvt. Ltd., Karachi, Pakistan); Lorsban^®^ (chlorpyrifos, 400 g/litre, 40 EC; Arysta Life Science Pakistan Pvt. Ltd., Karachi, Pakistan); Arrivo^®^ (cypermethrin, 100 g/litre, 10% EC; FMC United Pvt. Ltd., Lahore, Pakistan), Deltamethrin^®^ (deltamethrin, 25 g/ litre, 2.5% EC; Target Agro Chemicals, Lahore, Pakistan); Tracer^®^ (spinosad, 240 g/litre, 240 SC; Arysta Life Science, Pakistan Pvt. Ltd.), Karate^®^ (lambda-cyhalothrin 50g/litre, 5 EC; Syngenta Pakistan Ltd., Karachi, Pakistan) were used and phosphine was generated using aluminum phosphide tablets (Celphos 56%; Jaffer Brothers (Pvt.) Ltd., Lahore, Pakistan).

### Generation of phosphine (PH_3_) gas

The phosphine gas was generated using the FAO method [21]. The apparatus for generation of phosphine gas consisted of a 5 litre beaker, a collection tube (cylinder), an inverted funnel, aluminum phosphide tablets and muslin cloth. Gas collection tube was sealed from one side with an air-tight rubber stopper and then was filled with 5% sulphuric acid (H_2_SO_4_) solution. Half of the beaker was also filled with 5% H_2_SO_4_ solution. The gas collecting tube was placed carefully into the beaker over the inverted funnel in such a way that there is no loss of H_2_SO_4_ solution from the collection tube, while dipping into the beaker. Before generating phosphine gas all air was removed from the collection tube. Then aluminum phosphide tablets (wrapped in muslin cloth) were placed under inverted funnel. Phosphine gas was then collected in the gas collecting tube inverted over the funnel. As the funnel filled with generated gas, the level of solution dropped down. When the collecting tube was filled, 5 ml gas was sucked out with the help of an air tight syringe and was injected into sealed desiccators of known volume, then 50 ml of gas was taken out from the desiccators and injected into a phosphine meter to measure gas concentration allowing the required concentrations of phosphine gas to be obtained.

### Bioassays

#### Insecticides

From each population freshly molted F_1_ fourth-instar (L_4_) larvae of *R*. *ferrugineus* were challenged with the test insecticides. The F_1_ generation was obtained by mass mating of the field collected weevils and the weevils emerged from field-collected larvae and pupae. Toxicity bioassays were performed using artificial diet (Agar, brewer’s yeast, wheat germ, corn flour, ascorbic acid, benzoic acid, amino acid-vitamin mix, chloramphenicol and nipagin) [22]. Artificial diets were prepared by diluting the respective concentrations of commercial products in distilled water (mg litre^-1^of water) previously determined for each bioassay using Insecticide Resistance Action Committee (IRAC) Method No. 020 [23]. Accurate aqueous dilutions of each test chemical were prepared. For initial studies, eight widely spaced dose rates were used. Once a suitable range was identified a narrower range of six modified dose rates viz. 0, 20, 40, 60, 80 and 100 mg litre^-1^ were utilized which provided mortality between 5–95% of *R*. *ferrugineus* larvae. In the control treatment, diet was prepared using distilled water. A piece of artificial diet from each treatment was offered to ten freshly molted L_4_ larvae of *R*. *ferrugineus* individually in glass cups measuring (6×6 cm) with 40 mesh/inch screen lids for aeration and preventing the insects from escaping. All treatments were repeated three times and held at 27±2°C with 65±5% RH and a photoperiod of 12:12 (L: D) hours.

#### Phosphine (PH_3_)

FAO Method No. 16 [21] was used on 4^th^ instar larvae of *R*. *ferrugineus*. For each population, 10 larvae were placed individually, 1 in each of 10 glass cups and placed in 4 litre air tight glass boxes (serving as fumigation chambers) before phosphine fumigation. The cups had lids with tops made of 40 mesh/inch screen with 0.42-mm openings to permit fumigant entry and prevent from escaping. A small quantity of artificial diet (10 g) was added to each cup. The boxes were centrally equipped with a port on the metal screw on lid which was fitted with a rubber injection point which served as an entry point for phosphine. Before the lid was screwed onto the box, a rubber gasket was placed in it, and a thin layer of vacuum grease applied for a tight seal between the metal lid and the top edge to ensure gas was not leaking. Five phosphine concentrations (mg litre ^-1^) were measured in preliminary toxicity assays against 4^th^ instar larvae of *R*. *ferrugineus*. Another 4 litre box without any treatment served as control. The gas was introduced into each box containing *R*. *ferrugineus* larvae through the rubber septum by using a gas tight syringe after first removing an equivalent volume of air from the jar using a syringe. Two drops of water were added to each box using a syringe in order to maintain 70% RH inside the boxes. Boxes were then placed in an incubator maintained at 27±2°C. The boxes were opened 24 hours after application, concentration of phosphine in each jar was measured at the start and end of the exposure period, and an average concentration for the exposure period was calculated for each fumigation chamber. All the larvae from each treatment were placed on moistened filter paper and maintained at 27±2°C and 65±5% RH for a further 120 hours to allow recovery.

#### Statistical analysis

For insecticidal treatments, three days after the insects were removed from the insecticides treated-diet mortality counts were made in each treatment. However, for phosphine application individuals were exposed for 24h (+120 recovery hours) and then mortality was lumped together. Mortality data were corrected with Abbott's [24] formula to account for untreated (control) mortality. Corrected mortality data were subjected to Probit analysis [25] to calculate LC_50_ and LC_90_. LC_50_, LC_90_ and 95% fiducial limits were estimated for each insecticide at each location. Resistance ratios (RRs) were determined at LC_50_ by dividing the lethal concentration (LC) values of each insecticide by the respective LC values determined for the laboratory susceptible strain. Resistance ratios were categorized using the system of [26], “no resistance” (RR ≤ 1), “very low” (RR = 2–10), “low” (RR = 11–20), “moderate” (RR = 21–50), “high” (RR = 51–100), and “very high” (RR >100).

## Results

### Imidacloprid

**Fig 2:**
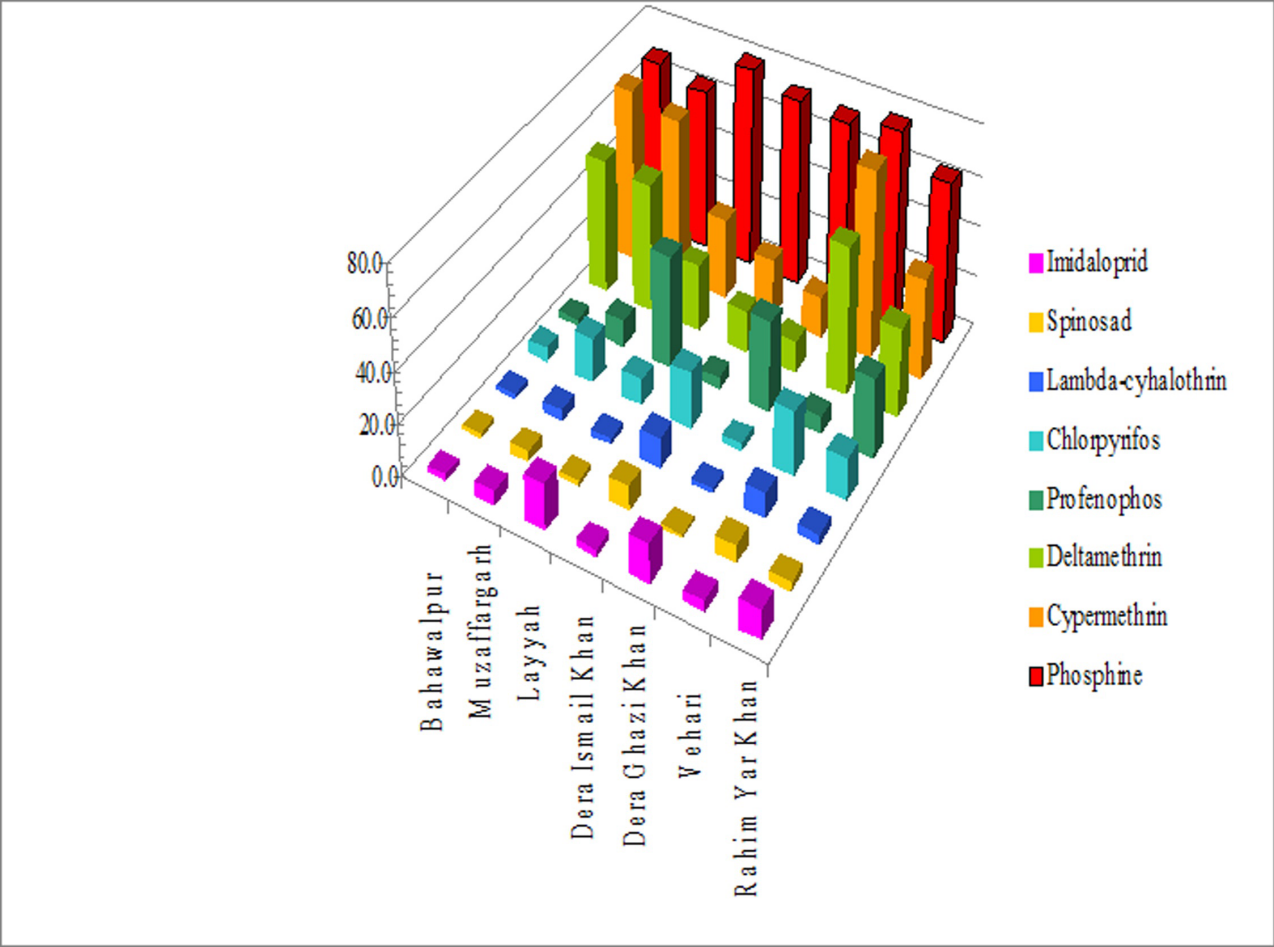
Resistance ratios (RR) of chemical insecticides and phosphine gas against a susceptible lab strain and field collected populations of *R*. *ferrugineus* from various localities in Punjab and KPK, Pakistan.

Resistance levels varied among the seven distinct field populations of *R*. *ferrugineus*. The degree of resistance varied from very low to low levels i.e. RR 2.10- to 17.68-fold for all the tested populations (Figs 1 and 2 and Table 1). A very low resistance level was recorded in Bahawalpur (2.10-fold), Dera Ismail Khan (2.86-fold), Vehari (4.20-fold) and Muzaffargarh (5.60-fold) populations at LC_50_ level. Low level of resistance was observed in populations of *R*. *ferrugineus* from Rahim Yar Khan (11.70-fold), Dera Ghazi Khan (15.12-fold) and Layyah (17.68-fold) at LC_50_ values.

**Table 1:** Resistance to commonly used insecticides and phosphine against susceptible strains and field collected populations of *R*. *ferrugineus*.

Insecticide	Localities	LC_50_ (mg litre^-1^) (95% fiducial limits)	LC_90_ (mg litre^-1^) (95% fiducial limits)	Slope	Resistance Ratio	Heterogeneity	χ^2^
Imidacloprid	Bahawalpur	16.59 (12.09–23.41)	250.87 (197.42–314.98)	1.12±0.18	2.10	1.23	0.23
	Muzaffargarh	44.22 (36.31–51.39)	669.08.16 (581.35.793.45)	1.42±0.19	5.60	1.46	0.06
	Layyah	139.52 (108.54–195.02)	2112.86 (1984.81–2358.44)	2.50±0.22	17.68	3.67	0.49
	Dera Ismail Khan	22.64 (18.76–29.78)	341.52 (285.16–459.49)	1.34±0.20	2.86	2.04	0.03
	Dera Ghazi Khan	119.37 (94.62–137.87)	1806.54 (1685.43–2132.38)	2.21±0.23	15.12	2.78	0.11
	Vehari	33.19 (26.14–42.81)	501.51 (435.32–651.32)	1.23±0.19	4.20	1.16	1.05
	Rahim Yar Khan	92.35 (79.37–117.69)	1397.43 (1258.15–1606.42)	1.36±0.21	11.70	2.61	2.25
	Laboratory	7.89 (5.81–12.25)	119.48(106.03–148.11)	0.74±0.14	-	2.48	0.47
Spinosad	Bahawalpur	7.98 (3.03–10.08)	128.22 (114.34–165.10)	0.92±0.16	2.03	2.17	1.02
	Muzaffargarh	17.26 (14.96–22.82)	276.34 (204.28–385.40)	1.14±0.18	4.40	1.23	0.07
	Layyah	9.32 (4.67–13.92)	150.22 (101.34–236.10)	1.06±0.15	2.37	0.67	0.00
	Dera Ismail Khan	38.30 (30.57–46.74)	620.75 (502.22–844.95)	1.42±0.19	9.77	2.02	1.76
	Dera Ghazi Khan	3.98 (2.80–5.55)	64.41 (42.16–97.84)	0.61±0.13	1.02	1.59	0.19
	Vehari	28.06 (24.87–33.91)	454.23 (357.81–648.35)	1.12±0.17	7.15	1.07	0.07
	Rahim Yar Khan	13.37 (10.75–17.21)	216.72 (141.38–321.18)	1.17±0.15	3.41	2.24	2.39
	Laboratory	3.92 (2.89–5.63)	63.56 (49.34–88.32)	0.69±0.14	-	0.78	1.06
Lambda-cyhalothrin	Bahawalpur	13.85 (10.51–18.83)	221.59 (165.41–352.82)	0.72±0.17	1.96	1.13	0.00
	Muzaffargarh	32.29 (26.54–42.12)	535.51 (448.32–679.32)	1.21±0.19	4.72	1.54	0.06
	Layyah	19.64 (15.31–24.44)	313.82 (217.46–416.10)	1.07±0.17	2.77	0.91	0.87
	Dera Ismail Khan	83.44 (73.39–95.72)	1347.16 (1081.84–1564.82)	1.61±0.21	11.88	2.08	1.62
	Dera Ghazi Khan	14.15 (10.97–19.21)	228.76 (151.39–318.30)	0.72±0.15	2.01	0.42	0.04
	Vehari	64.76 (56.34–71.72)	1045.11 (803.22–1281.35)	1.41±0.21	9.21	1.19	0.00
	Rahim Yar Khan	26.44 (20.31–75.63)	425.93 (319.32–620.47)	1.33±0.20	3.76	1.75	1.38
	Laboratory	7.02 (5.21–11.27)	113.48(102.03–130.10)	0.90±0.15	-	2.27	0.06
Chlorpyrifos	Bahawalpur	44.21 (37.57–53.40)	611.92 (499.40–881.55)	1.22±0.19	5.45	1.52	1.04
	Muzaffargarh	145.36 (117.57–174.38)	2011.35 (1861.65–2351.88)	2.42±0.21	17.91	1.05	2.21
	Layyah	86.42 (73.70–105.08)	1195.43 (996.15–1440.42)	1.37±0.20	10.65	0.94	0.00
	Dera Ismail Khan	177.25 (145.86–197.65)	2452.54 (2275.47–2865.36)	2.31±0.21	21.84	1.21	0.08
	Dera Ghazi Khan	24.61 (20.48–30.22)	340.55 (249.42–481.64)	1.72±0.19	3.03	1.66	3.10
	Vehari	198.52 (146.54–237.53)	2747.67 (2505.43–3018.38)	2.55±0.20	24.47	0.87	1.93
	Rahim Yar Khan	129.64 (97.54–182.38)	1794.75 (1598.89–2121.52)	2.31±0.18	15.98	1.34	0.04
	Laboratory	8.11 (4.01–11.92)	112.29 (78.34–208.10)	0.97±0.16	-	1.20	1.37
Profenophos	Bahawalpur	23.31 (19.18–27.71)	307.01 (261.52–377.90)	1.25±0.18	2.65	1.27	1.13
	Muzaffargarh	92.19 (78.12–115.08)	1220.43 (1161.15–1383.42)	1.30±0.21	10.50	0.79	0.45
	Layyah	387.26 (330.65–424.59)	5125.41 (4871.21–5561.27)	3.22±0.23	44.1	1.65	2.78
	Dera Ismail Khan	40.20 (32.98–46.87)	531.87 (414.23–680.45)	1.41±0.18	4.57	1.26	0.00
	Dera Ghazi Khan	309.37 (286.17–353.53)	4093.08 (3814.56–4405.82)	2.53±0.18	35.22	1.07	1.04
	Vehari	60.64 (52.34–67.72)	801.21 (719.22–1029.35)	1.41±0.17	6.90	0.34	2.72
	Rahim Yar Khan	266.28 (224.23–305.29)	3522.54 (3368.83–3917.76)	2.56±0.20	30.31	1.86	3.57
	Laboratory	8.78 (4.44–12.35)	116.22 (90.45–174.59)	0.99±0.16	-	2.28	2.34
Deltamethrin	Bahawalpur	348.28 (296.65–374.59)	5729.41 (5204.21–6480.27)	3.61±0.23	53.81	1.43	1.12
	Muzaffargarh	332.43 (283.65–365.59)	5469.41 (5108.21–5911.27)	3.36±0.21	51.37	1.22	0.45
	Layyah	173.29 (144.54–199.65)	2849.54 (2617.47–3227.36)	2.31±0.20	26.76	0.89	0.01
	Dera Ismail Khan	106.34 (96.12–121.05)	1748.22 (1553.08–2092.29)	1.73±0.19	16.42	0.67	3.23
	Dera Ghazi Khan	83.54 (73.24–92.11)	1373.16 (1202.84–1591.82)	1.54±0.17	12.90	1.39	0.89
	Vehari	375.95 (329.65–411.59)	6172.41 (5728.21–6798.27)	3.52±0.23	57.97	1.81	0.06
	Rahim Yar Khan	223.41 (194.36–284.66)	3675.76 (3339.78–4105.55)	2.72±0.21	34.52	2.87	0.00
	Laboratory	6.47 (4.97–9.12)	106.48(93.03–122.13)	0.71±0.14	-	1.34	0.56
Cypermethrin	Bahawalpur	503.12 (454.22–583.48)	8127.65 (7875.63–8587.39)	3.72±0.23	69.49	0.67	3.49
	Muzaffargarh	565.54 (391.43–605.66)	7519.76 (7256.32–7991.18)	3.83±0.22	64.29	1.64	1.07
	Layyah	225.39 (201.57–267.29)	3638.02 (3425.25–4058.32)	2.59±0.20	31.11	2.01	0.06
	Dera Ismail Khan	163.65 (139.23–204.32)	2641.58 (2470.43–2911.43)	2.48±0.19	22.59	1.83	2.34
	Dera Ghazi Khan	115.18 (94.12–128.11)	1859.64 (1715.76–2119.64)	2.16±0.21	15.89	1.23	0.00
	Vehari	534.47 (489.22–622.48)	8633.65 (7247.63–9211.39)	3.65±0.23	73.82	0.52	0.02
	Rahim Yar Khan	255.42 (233.23–311.23)	4609.54 (4266.83–5165.76)	2.81±0.21	39.41	1.96	2.83
	Laboratory	7.24 (5.39–11.23)	116.96(103.77–134.12)	0.71±0.19	-	1.02	1.74
Phosphine	Bahawalpur	4008.70 (3280.23–4955.42)	51177.95 (43719.73–59088.76)	5.46±0.27	68.76	0.85	1.04
	Muzaffargarh	3718.37 (3011.23–4604.56)	47679.37 (42165.65–55248.54)	5.82±0.24	63.78	2.54	1.56
	Layyah	4632.51 (3846.43–5517.72)	59402.62 (51484.42–64804.32)	6.37±0.34	79.46	1.27	0.06
	Dera Ismail Khan	4293.21 (3618.43–5180.41)	55048.84 (50342.23–62268.54)	6.20±0.30	73.64	1.51	1.12
	Dera Ghazi Khan	4214.50 (3529.43–5065.54)	54039.67 (5045534–621180.23)	5.82±0.28	72.29	2.34	0.00
	Vehari	4448.33 (3750.43–5309.52)	57038.53 (50118.76–63308.37)	6.02±0.31	76.3	0.67	0.78
	Rahim Yar Khan	3678.14 (2972.23–4545.56)	47163.47 (42540.65–54540.84)	4.83±0.23	63.09	1.02	1.89
	Laboratory	58.3 (52.45–64.54)	747.56 (692.54–894.89)	1.48±0.21	-	1.28	2.45

### Spinosad

Very low level (1.02- to 9.77-fold) resistance ratios were recorded among different field populations of *R*. *ferrugineus* (Fig 2 and Table 1).

### Lambda-cyhalothrin

Very low to low levels (1.96- to11.88-fold) RR were recorded (Fig 2 and Table 1).

### Chlorpyrifos

Very low and low to moderate levels (3.03- to 24.47-fold) RR were recorded among tested populations of *R*. *ferrugineus* (Fig 2 and Table 1).

### Profenophos

Very low and low to moderate levels (RR 2.65- to 44.10-fold) were recorded for profenophos among different field populations of *R*. *ferrugineus* (Fig 2 and Table 1).

### Deltamethrin

Low and moderate to high levels (RR 12.90- to 57.97-fold) of resistance were recorded among different field populations of *R*. *ferrugineus* (Fig 2 and Table 1).

### Cypermethrin

Low and moderate to high levels (RR 15.89- to 73.82-fold) of resistance were recorded among different field populations of *R*. *ferrugineus* (Fig 2 and Table 1).

### Phosphine

High levels of resistance were recorded against phosphine in all the tested populations of *R*. *ferrugineus*, ranging from 63.09- to 79.46-fold (Fig 2 and Table 1) with very little difference between populations.

## Discussion

Knowledge of the resistance status of pests is important for researchers to guide the farming community in combating pest problems. It could become helpful for growers to reduce or suspend the use of particular chemicals in their plantings. These seven areas we worked on are the major date producing areas of Pakistan and contribute the major share of the country’s date production and have a history of *R*. *ferrugineus* infestation stretching back almost 100 years [27]. To combat this pest, farmers have mainly used conventional insecticides and fumigants, particularly phosphine.

This is the first report of an insecticide-resistance investigation in *R*. *ferrugineus* in Pakistan. Populations of *R*. *ferrugineus* were established from field collections and all strains exhibited different susceptibility levels to all tested insecticides through dose-mortality bioassays, except for Dera Ghazi Khan strain for spinosad. LC_50_ with overlapping confidence intervals compared to the susceptible strain. In our study a laboratory population was considered a reference strain which exhibited no resistance to the tested chemicals which was very close to [28]. Among the tested chemicals spinosad and lambda-cyhalothrin were the most effective and revealed a very low level of resistance and LC_50_ in any tested population. The insecticides fed in an artificial diet to larvae caused mortality in a dose-dependent manner. The most consistent resistance across seven populations was recorded for deltamethrin and cypermethrin. High level of resistance to phosphine was observed in all populations. The current study provides a base line of resistance to cypermethrin, deltamethrin and phosphine against *R*. *ferrugineus*. In the strains examined low and moderate to high level of resistance was probably due to the excessive ineffective use of these chemical insecticides without knowing the chemistry and mode of action to manage *R*. *ferrugineus* in Pakistan.

The cryptic habit of *R*. *ferrugineus* facilitates almost year-round activity in date palm plantations which has forced the farmers to expose field populations to different chemical insecticides and fumigants in order to successfully control pest infestations. The repeated application of these chemical insecticides could then have led to the development of genetic resistance in *R*. *ferrugineus* populations. Resistance to cypermethrin and deltamethrin is common among arthropod pests worldwide. In Pakistan resistance against these commonly used insecticides in various crop pests such as in *Spodoptera exigua* (Hübner) (Lepidoptera: Noctuidae), *Brevicoryne brassicae* (L.) (Hemiptera: Aphididae), *Spodoptera litura* (Fabricius) (Lepidoptera: Noctuidae), *Helicoverpa armigera* (Hübner) (Lepidoptera: Noctuidae) and *Bemisia tabaci* (Gennadius) (Homoptera: Aleyrodidae) has been reported by many scientists [28, 29, 30, 31, 32, 33, 34]. So far only one published record of cypermethrin resistance against *R*. *ferrugineus* is reported, from Saudi Arabia [35]. Resistance to phosphine is not surprising because deployment of phosphine in the form of aluminum phosphide tablets is common practice among date palm farming community in Pakistan. Resistance to phosphine is common in stored grain insets such as *Tribolium castaneum* (Herbst.) (Coleoptera: Curculionidae), *Rhyzopertha dominica* (F.) (Coleoptera: Bostrichidae) and psocid species (Liposcelididae: Psocoptera) worldwide e.g. Opit et al. [36] reported 119 fold and 1500 fold resistance in *T*. *castaneum* and *R*. *dominica* strains respectively, collected from Oklahoma, USA. Many other researchers have reported phosphine resistance in stored product pests from all over the world [37].

It is advised not to use cypermethrin and deltamethrin against this pest and alternative chemicals such as spinosad and lambda-cyhalothrin should be utilized in control strategies. So far, there is no understanding of the genetics of resistance in this species or of any cross-resistance pattern. However, multiple resistances to conventional and new chemical insecticides have been recorded previously in Pakistan in other species, with some incidence of cross-resistance within insecticide classes [20]. In Pakistan it is a common practice to mix conventional and newer insecticides to control insect pests of economically important crops; therefore, it would be premature to conclude that a cross-resistance exists between these compounds. Spinosad and lambda-cyhalothrin possesses high efficacy against the damaging larval stage of *R*. *ferrugineus* in laboratory assays, as in the present work [14], and their inclusion in control programs using the most effective application method would be worth considering. Treatment strategies with chemical insecticides which include spinosad in control programs with changed pattern would be worth considering. Moreover, integrated use of microbial control agents with newer insecticides with novel modes of action could successfully replace insecticides to which resistance has developed in field control programs.
